# Effects of fecal microbiota transplantation in subjects with irritable bowel syndrome are mirrored by changes in gut microbiome

**DOI:** 10.1080/19490976.2020.1794263

**Published:** 2020-09-29

**Authors:** Rasmus Goll, Peter Holger Johnsen, Erik Hjerde, Joseph Diab, Per Christian Valle, Frank Hilpusch, Jorunn Pauline Cavanagh

**Affiliations:** a Research Group of Gastroenterology and Nutrition, Department of Clinical Medicine, UiT the Arctic University of Norway, Tromsø, Norway; b Department of Gastroenterology, Division of Internal Medicine, University Hospital of North Norway, Tromsø, Norway; c Department of Internal Medicine, University Hospital of North Norway, Harstad, Norway; d Institute of Chemistry, UiT the Arctic University of Norway, Tromsø, Norway; e Natural Products and Medicinal Chemistry Research Group, Department of Pharmacy, UiT the Arctic University of Norway, Tromsø, Norway; f Sjøkanten Legesenter, Harstad, Norway; g Pediatric Infections Group. Department of Pediatrics, University Hospital of North Norway, Tromsø, Norway; h Pediatric Infections Group. Department of Clinical Medicine, UiT the Arctic University of Norway, Tromsø, Norway

**Keywords:** Irritable bowel syndrome, fecal microbiota transplantation, engraftment, metagenomic sequencing, taxonomic profile, diversity, growth rate, functional features

## Abstract

Irritable bowel syndrome (IBS) is a common disorder of the lower gastrointestinal tract. The pathophysiology is far from settled, but a gut microbial dysbiosis is hypothesized to be a contributing factor. We earlier published a randomized double-blind placebo-controlled clinical trial on fecal microbiota transplantation (FMT) for IBS – the REFIT trial. The present data set describes the engraftment and includes participants from the study who received active FMT; 14 participants with effect of FMT (*Effect*) and 8 without (*No effect*). Samples were collected at baseline, after 6 and 12 months. Samples from the transplants (*Donor*) served as a comparator. In total 66 recipient samples and 17 donor samples were subjected to deep metagenomic sequencing, and taxonomic and functional analyses were performed. Alpha diversity measures showed a significantly increased diversity and evenness in the IBS groups compared to the donors. Taxonomic profiles showed higher relative abundance of phylum Firmicutes, and lower relative abundance of phylum Bacteroidetes, compared to donors at baseline. This profile was shifted toward the donor profile following FMT. Imputed growth rates showed that the resulting growth pattern was a conglomerate of donor and recipient activity. Thirty-four functional subclasses showed distinct differences between baseline samples and donors, most of which were shifted toward a donor-like profile after FMT. All of these changes were less pronounced in the *No effect* group. We conclude that FMT induces long-term changes in gut microbiota, and these changes mirror the clinical effect of the treatment. The study was registered in ClinicalTrials.gov (NCT02154867).

## Background

Irritable bowel syndrome (IBS) is a functional gut disorder characterized by abdominal pain or discomfort associated with abnormal frequency and consistency of bowel movements. IBS presents as one of the three phenotypes: IBS with diarrhea (IBS-D), constipation (IBS-C), or mixed (IBS-M). IBS is a very common complaint with an estimated global prevalence of 11.2%, but higher in Northern Europe, and more frequent among young women than in men.^1^

A key challenge in IBS research is that the pathophysiology is largely unknown, and remains controversial. Interaction between host and gut microbiota is considered a new clinical frontier. Experimental evidence and theoretical considerations imply that disturbances in this interaction may have multiple effects as diverse as obesity, metabolic syndrome, liver cirrhosis, inflammatory bowel disease, and colorectal cancer.^2^ The current hypothesis on IBS pathophysiology focuses on the interaction between gut microbiota and host factors such as immune system activation, altered neuroendocrine signaling, and gut mucosal barrier function.^3^ This hypothesis is supported by the notion that IBS-like traits can be observed in rats following fecal transplant from IBS patients.^4^

Both diet regimens (low intake of fermentable oligo-, di-, and monosaccharides and polyols (low-FODMAP); low fructose; gluten-free), food supplements (soluble fiber, peppermint oil), probiotics, and pharmacologic treatments (e.g., prostaglandin analogs, 5HT_3_ antagonists, antispasmodics, and antibiotics) are available treatments of IBS. However, the drugs with best documentation show low to moderate effect, and the dietary interventions have not been evaluated extensively.^3,5^ In Europe, the agreed intervention of choice is in general dietary interventions, like low FODMAP.^6^ Documentation of its efficacy is, however, relatively poor, ^7^ and the theoretical framework is not completely understood. Several mechanisms of action have been suggested including both microbial shifts in profile and metabolic output, and changes in the intestinal regulatory systems like serotonin-producing cells (reviewed by Staudacher and Whelan^8^).

It has been hypothesized that a *dysbiosis* of the gut flora is an important part of the pathophysiology of IBS.^9,10^ This is supported by the finding of a distinctly different metagenomic stool profile in a large case-control study on IBS vs healthy controls (and inflammatory bowel disease).^11^ Consequently, altering the gut microbiome by fecal microbiota transplantation (FMT) has been suggested as a possible treatment option in IBS.^12–14^

Our previously published REFIT trial provided the first proof of concept for FMT in IBS patients.^15^ It included 90 IBS patients in a randomized double-blind placebo-controlled clinical trial of donor FMT vs. placebo in a 2:1 randomization. Patients with IBS-D or IBS-M defined by the ROME III criteria, scored as moderate to severe according to the IBS severity scoring system (IBS-SSS), were enrolled locally by general practitioners in northern Norway. The donor feces was freshly processed, and was used the same day, or frozen for later use. The participants’ own preprocessed and frozen feces served as placebo. The transplant was administered by a colonoscope to the cecum. The main outcome measure was self-rated clinical effect assessed by the IBS-SSS (reduction of >75 points). The trial showed significant clinical effects 3 months after administration of FMT, with very few and minor self-limited adverse effects until 1 year post-FMT.

However, the effect of FMT in IBS is contested, as not all studies agree on its efficacy.^16^ The mode of delivery may be a key issue, as classic FMT shows promise, while capsule delivery does not.^17^ In order to understand this observed variability, it is necessary to characterize the intervention of classic FMT in detail.

Here, we provide a data set generated by deep metagenomic sequencing of fecal samples collected before and after FMT during the REFIT trial.

## Results

Stool was collected at three time points from the 83 participants who completed the REFIT study. The present data set was constructed to investigate the effects of engraftment of the donor transplant; hence, we picked only participants from the active treatment group who received donor transplants. Only 22 participants with complete sample sets were available due to low compliance in delivery of fecal samples after the treatment. Of these, 14 had good effect (termed *Effect*), and 8 had none or only a transient effect of the intervention (termed *No effect*). Seventeen samples from the transplants (termed *Donors*) used for treatment of the 22 participants, from two individual donors, acted as comparator group in the present setup. The baseline characteristics for the selected study participants are presented in Table 1. At baseline, no significant differences in patient characteristics were detected between the IBS groups.

**Table 1.** Baseline characteristics. Key characteristics of the participants in the present data set. *P* values generated by Mann–Whitney for continuous variables, and Fischer’s Exact Test for categorical variables.

	*Donor* (n = 2)	*Effect* of FMT (n = 14)	*No effect* of FMT (n = 8)	P
Age mean (range)	18	47 (19–69)	49 (30–70)	0.87
Sex F/M	0/2	10/4	6/2	1.00
Body mass index (kg/m2; median (range))	n/a	24.8 (21.3–41.2)	25.7 (19.6–33.6)	0.94
IBS-D/IBS-M	n/a	9/5	4/4	0.66
IBS-SSS mean (95%CI)	n/a	271 (239–303)	259 (210–308)	0.62
Years w IBS mean (95%CI)	n/a	16 (9–23)	14 (1–27)	0.48
FODMAP intake mean g/day (95%CI)	n/a	13 (6–20)	11 (2–20)	0.71
Fresh/frozen transplant	n/a	7/7	6/2	0.38

### Alpha diversity

**Figure 1.**
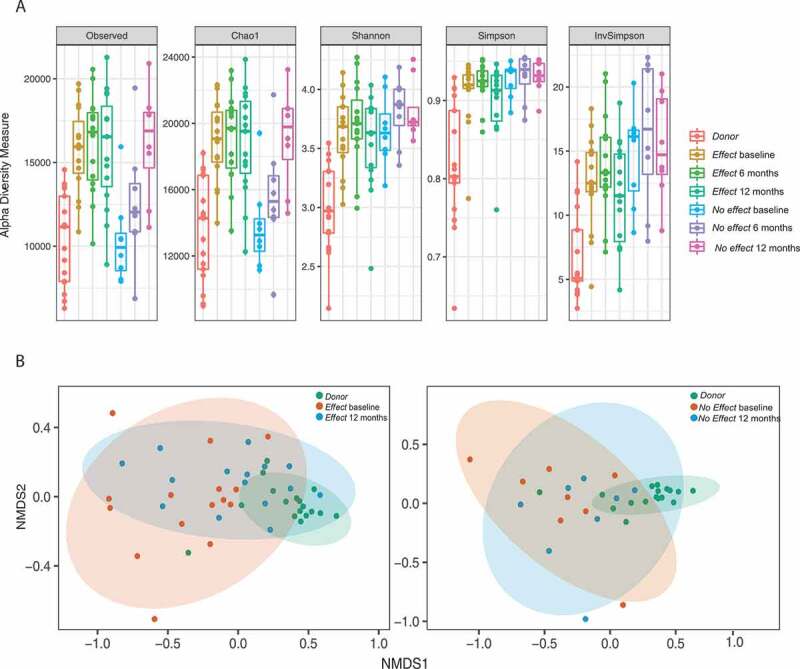
Panel A: Box plots with alpha diversity estimates where each sub-panel shows a different type of estimator. The colors group the samples into seven groups, and each point represents the richness estimate per sample. The data was filtered so that taxa occurring < five times in at least 20% of the samples were excluded from the analysis. Panel B: nMDS ordination plots using Bray-Curtis distance between samples. Comparisons of the *Effect* group at baseline, after 12 months and the donor samples are visualized separate from the *No effect* group at baseline, after 12 months and the donor samples. The data were filtered so that taxa with a mean fractional abundance < 10^−5^ were excluded from the analysis.

Measures of diversity and evenness are presented in Figure 1(a). The richness (Chao1 and Observed) was significantly higher in the *Effect* group than in the *Donor*s at all time points (*P* = .0002; 0.0002; 0.0012 for baseline, 6 months, and 12 months, respectively, for Observed). The *No effect* group had significantly lower richness at baseline (*P* = .0049 for Observed) compared to the *Effect* group, with values comparable to the *Donors* (*P* = 1.0000 for Observed). At 12 months, the richness in the *No Effect* group increasingly resembled that of the *Effect* group (*P* = 1.0000 for Observed). However, looking at diversity measures that include evenness (Shannon and Simpson), both IBS participant groups showed higher diversity at baseline compared to the *Donor*s (*P* = .0026 and 0.0046 by Simpson index for *Effect* and *No effect* groups, respectively), and these measures did not change significantly with time.

### Beta diversity

Non-metric multidimensional scaling (NMDS) was performed on beta diversity in the fecal samples from the *Donor, Effect*, and *No Effect* groups prior to FMT and after 12 months (Figure 1(b), left panel). A clear separation between *Donor* and *Effect* samples was found at both time points. While the microbial composition of fecal samples from the *Effect* group, after 12 months, clustered closer to the *Donor* samples, the *No effect* samples did not show any separation in clustering between the sampling time points (Figure 1(b), right panel).

### Relative taxonomic abundances

**Figure 2.**
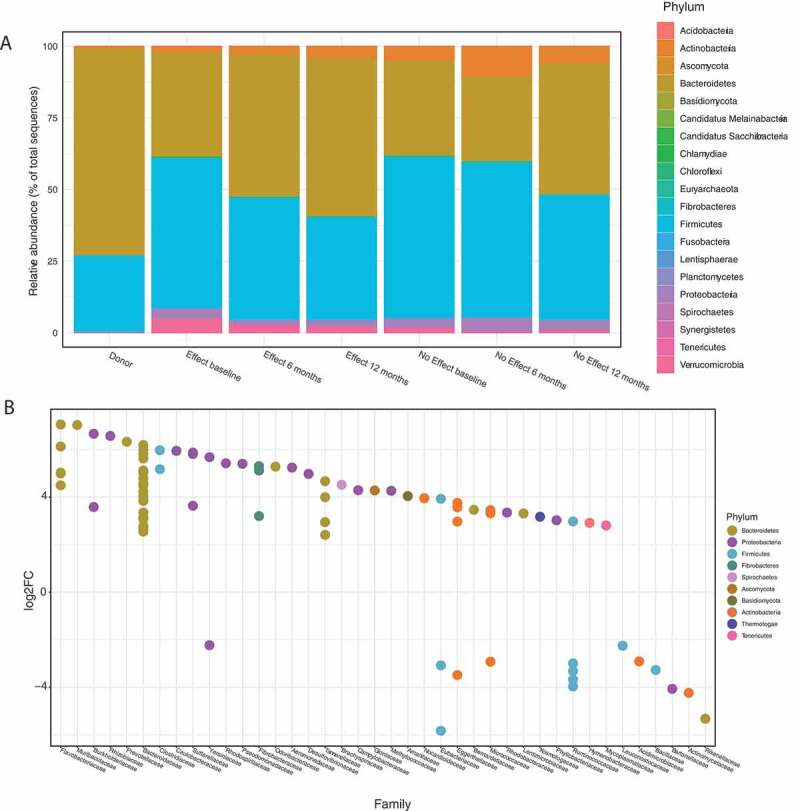
Panel A: Relative abundance of the most prevalent phyla for the seven groups. The data were filtered so that taxa not seen more than 5 times in at least 20% of the samples in the total dataset were removed. Following this, only taxa with a mean greater than 10^−5^ (fractional abundance > 0.00001) were kept before agglomeration at phylum level. Panel B: Differential abundance calculated with DESeq2 at phylum and family level, in the *Effect* group, 12 months vs. baseline. Positive log2FC indicate enriched taxa after FMT, negative log2FC indicate decreased taxa after FMT. Families indicated on the X-axis are colored according to phyla. FDR cut off for inclusion in the plot was < 10^−4^.

The taxonomic profiles of the *Donor* and IBS patient samples were widely different (Figure 2(a); only phylum is shown). The *Donor* samples had a relatively higher abundance of Bacteroidetes than the patient samples, and lower abundance of Firmicutes and Actinobacteria. The relative abundance profiles changed over the sampling period in both the *Effect* and *No effect* groups, becoming more similar to the *Donor* profile with increased abundance Bacteroidetes and a reduction of Firmicutes. For the *No effect* group, however, the 6 month samples, in particular, had relatively low abundance of Bacteroides and relatively high abundance of Actinobacteria.

### Differential abundance

In order to identify taxa that changed significantly in the *Effect* group, from baseline to 12 months, the data set was subjected to an unsupervised differential abundance analysis using differential gene expression based on the negative binomial distribution (DESeq2) (Figure 2(b), S1). At baseline, prior to FMT, species belonging to the phyla Firmicutes, Actinobacteria, Proteobacteria (seven different species), and Verrumicrobia were more abundant. Twelve months after FMT a higher abundance of species belonging to the phyla Bacteroidetes and Proteobacteria was found (23 different species) of which species belonging to the Bacteroidetes was particularly more abundant.

In total, 170 species showed a significantly different abundance in response to FMT (false discovery rate (FDR) <0.01 and log2fold change <-2 or >2) (Table S1); of these, 128 species increased in abundance and 42 decreased in abundance after FMT. Twenty-four of the 36 species belonging to the Firmicutes phylum showed a reduced abundance following FMT (66% of Firmicutes; 14% of 170). In phylum Bacteroidetes, 64 of 67 species increased in abundance after FMT (96% of Bacteroidetes; 38% of 170). Of species known to be involved in the production of short-chain fatty acids (SCFA), 3 of 4 *Eubacterium sp*., 0 of 2 *Clostridium sp*., 11 of 12 *Ruminococcus sp*., 2 of 2 *Klebsiella sp*., and 2 of 2 *Lactobacillus sp*. had a decreased abundance after FMT. However, considering baseline counts of these species, the resulting SCFA production capacity (based on mean baseline counts by fold change) increased by a factor 6.7 after FMT. *Akkermansia muciniphila* had a significantly reduced abundance at 12 months.

### Multivariate regression model of the taxonomic analysis

**Figure 3.**
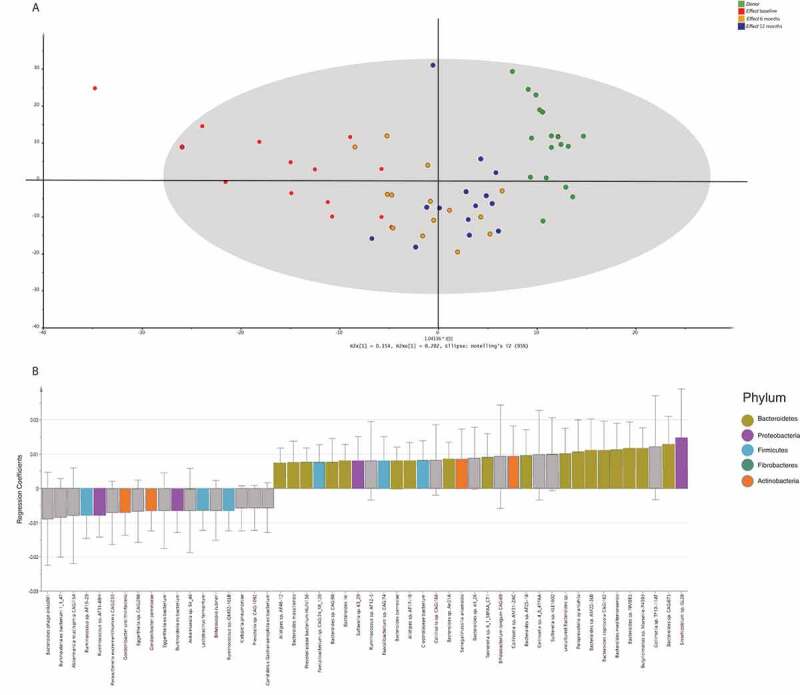
Multivariate regression analysis of the bacterial species. Each sample was labeled according to the corresponding study group. Panel A: This t1/t2-score plot of the orthogonal partial least squares projection to latent structures (OPLS) model (one predictive component and one orthogonal component) was built from the bacterial species composition in stool samples taken from the *Donors*, and from the *Effect* group at 3 time points: baseline, 6, and 12 months after FMT. The performance parameters R2Xcum, R2Ycum and Q2cum were 0.36, 0.61 and 0.32, respectively. Panel B: The top 50 bacterial species ranked by regression coefficients pertaining to the predictive components. To make the coefficients readily comparable, the independent variables for different taxa were scaled and centered prior to the analysis. The error bars indicate the confidence intervals of the coefficients. The coefficient is considered significant (above noise level), when the confidence interval does not include zero. Significant features are color-coded according to phylum.

The orthogonal partial least squares projection to latent structures (OPLS) analysis showed that the taxonomic profile in the *Effect* group moved toward the *Donor* profile over time after FMT treatment. At 12 months, however, the profiles were still clearly distinct. Notably, the most profound change was observed between baseline and 6 months after the FMT treatment, Figure 3(a). From the OPLS model, a shortlist of the 50 most important species affecting the shift in taxonomy profile over time was generated based on regression coefficients, Figure 3(b). The list shows that 7 species decreased after treatment (3 Firmicutes, 2 Actinobacteria, and 2 Proteobacteria), and 25 increased (18 Bacteroidetes, 3 Firmicutes, 2 Proteobacteria, and 2 Actinobacteria).

### Growth rate dynamics

**Figure 4.**
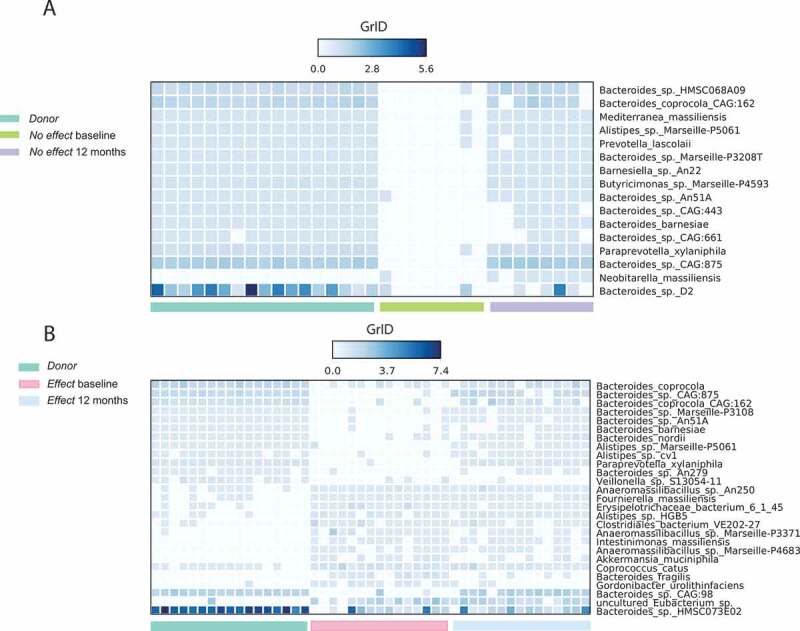
Growth rate score (GRiD) of the most frequent occurring species for samples in: (a) *No effect* group at baseline (green), after 12 months (purple) and the *Donor* samples (cyan); and (b) *Effect* group at baseline (pink), after 12 months (blue) and the *Donor* samples (cyan). GRiD score > 1 is an indication of bacteria in the growth phase, while GRiD score < 1 indicates bacteria in stationary or lag phase. The data were filtered so that species occurring < 15 times in < 30 samples were excluded from the analysis. FDR cut off for inclusion in the heat map was < 0.00005.

Growth rates for bacterial species identified in the *Donor* samples, and from the *Effect* group prior to treatment and after 12 months, were estimated using the growth rate index (GRiD).^18^ In total, GRiD reported 959 species for the 47 samples. After filtering of low-frequent species (occurring <15 times in <30 samples), and including only species with an FDR < 0.00005, we identified 27 species that displayed a significantly different growth rate in the three groups, Figure 4. The majority of these species were in the lag or stationary phase prior to FMT but were in exponential growth phase both in the *Donor* samples and in samples taken after FMT. The largest group of species found were belonging to the phylum Bacteroidetes (15/27), followed by different species of the phylum Firmicutes (8/27), among these *Coprococcus catus*, a known butyrate producer.

### Functional analysis

**Figure 5.**
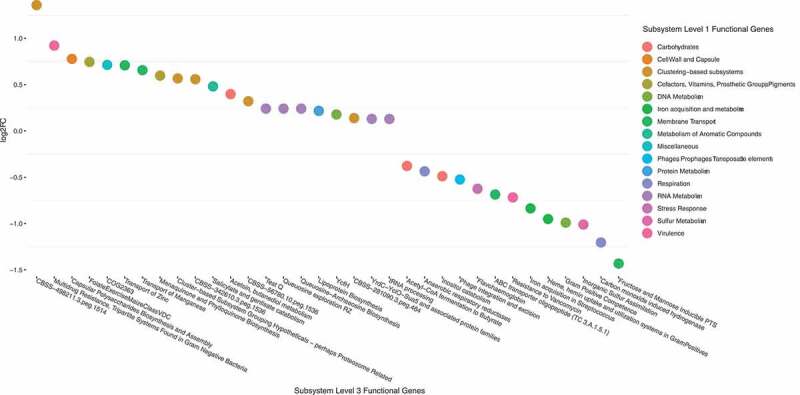
Differential abundance of functional subsystems with absolute abundance > 0.01% in the *Effect* group, 12 months vs. baseline. Positive log2FC indicate enriched subsystems after FMT, negative log2FC indicate decreased subsystems after FMT. Subsystem level 3 indicated on the X-axis are color-coded according to level 1 classification. FDR cut off for inclusion in the plot was < 0.05.

In total, 34 functional subsystems (SEED; please see the Methods section) showed a significant differential abundance in samples from the *Effect* group after 12 months, compared to the corresponding baseline samples (Figure 5). Thirteen functional subsystems were significantly less abundant after FMT (13/34 – 38.2%), while 21 were significantly more abundant after FMT. The subsystem *Fructose and Mannose inducible PTS*, involved in sugar transport, showed the largest decrease in abundance of all subsystems after FMT. The *Inorganic sulfur assimilation subsystem* and the *Heme and hemin uptake and utilization systems in Gram positives* were both significantly less abundant. *Acetyl-CoA fermentation to butyrate* was less abundant at baseline, however not significantly so. Two functional subsystems involved in *Menaquinone, K2 biosynthesis*, and two subsystems involved in uptake of Zinc and Manganese in addition to the *tetrahydrofolate biosynthesis pathway* were significantly more abundant at 12 months post-FMT. The subsystem *CBSS-498211.3.peg.1514*, with function unknown, showed the largest change of all at 12 months post-FMT.

**Figure 6.**
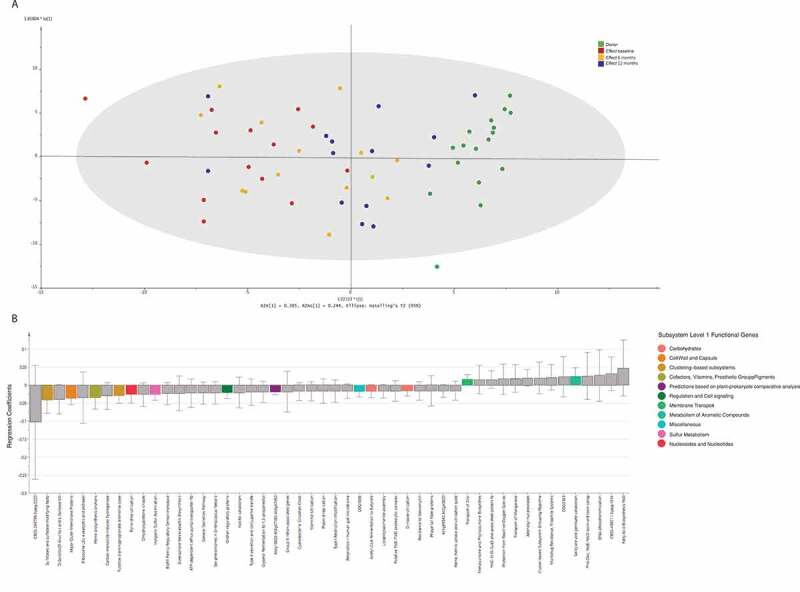
Multivariate regression analysis of the functional groups. Each sample was labeled according to the corresponding study group. Panel A: This t1/t2-score plot of the orthogonal partial least squares projection to latent structures (OPLS) model (one predictive component and one orthogonal component) was built from functional groups in stool samples taken from the *Donors*, and from the *Effect* group at 3 time points: baseline, 6, and 12 months after FMT. The performance parameters R2Xcum, R2Ycum and Q2cum were 0.55, 0.52 and 0.30, respectively. Panel B: The top 50 functional groups ranked by regression coefficients pertaining to the predictive components. To make the coefficients readily comparable, the independent variables for different functional groups were scaled and centered prior to the analysis. The error bars indicate the confidence intervals of the coefficients. The coefficient is considered significant (above noise level), when the confidence interval does not include zero. Significant features are color-coded according to level 1 classification.

We then calculated individual differences in relative abundance between *Donors* and recipients (baseline sample vs. *Donor* sample used for each participant), as well as individual changes (12 months vs. baseline) in each of the 34 functional classes, for each participant. The baseline differences between *Donors* and recipients were quite similar in *Effect* and *No effect* groups (Figure S2, panel A). At 12 months, however, convergence toward the *Donor* profile was more profound in the *Effect* group than in the *No effect* group (Figure S2, panel B). Notably, *Carbohydrates* was the only functional class with a positive change in the *Effect* group, which could not be found in the *No effect* group. However, this difference was not significant. A further sub-analysis focused on the *Carbohydrates* cluster revealed subtle differences in systems involved in *D-Galacturonate and D-Glucuronate utilization, D-ribose utilization, L-rhamnose utilization, Lactose and Galactose uptake and utilization*, and *Lactose utilization* (Figure S3). Next, a multivariate regression model of the functional level 3 classes was built. Overall, the OPLS model (Figure 6(a)) showed the same movement from baseline toward *Donor* profile during the time course, though not as clearly as seen in the taxonomic regression model (Figure 3(a)). Eleven functional subsystems showed significant changes following FMT, these were pathways associated with *Acetyl-CoA fermentation, inorganic sulfur assimilation, transport of Zinc*, and *heme biosynthesis orphans* (Figure 6(b)).

## Discussion

We have performed deep metagenomic sequencing of a selection of samples from our previously published randomized clinical trial on FMT vs placebo for non-constipated IBS. We found that alpha diversity was higher in stool from IBS subjects than in stool from the *Donor*s. For subjects who experienced an effect of the treatment, a trend of convergence toward the *Donor* microbiome profile after FMT was shown; this trend was however less clear for subjects with no treatment effect. This trend was seen both on the taxonomic and on the functional level by the dimension reduction analysis and was even more clearly demonstrated by the multivariate regression (OPLS) models. Selected features on the phyla level, such as the fraction of Bacteroidetes and Firmicutes, showed the same trend (Figure 1(a) and (b)).

### Taxonomy

Compared to *Donor* samples, the baseline profile of IBS patients found in the present data set shows a relative dominance of Firmicutes over Bacteroidetes, while *Donor* samples show the opposite pattern. This finding supports an earlier report using similar methodology, ^11^ and some studies using 16S ribosomal ribonucleic acid (16S rRNA) sequencing.^19,20^ However, our Firmicutes:Bacteroidetes result is challenged by other studies where 16S rRNA sequencing demonstrates the exact opposite balance in IBS,^21–23^ or no distinct IBS profile.^24^ Factors such as race, regional diet, and methodology may have had an impact on the taxonomic profiles reported.^25^ A recent review on 16S rRNA sequencing data addresses the Firmicutes:Bacteroidetes schism, as well as the question of diversity, concluding that most data sets support the increased Firmicutes:Bacteroidetes ratio in IBS, while a lower alpha diversity is a more prevalent finding in IBS.^26^ Further support for the hypothesis that microbiota composition may be a key feature in IBS pathophysiology can be drawn from our longitudinal data showing increasing relative abundance of Bacteroidetes, and decreasing relative abundance of Firmicutes during the time course of the study. Thus, the microbiome profile change mirrors the clinical effect observed after FMT.

Moreover, the present data set showed that, based on taxonomic data, the short-chain fatty acid (SCFA) producing species such as *Ruminococcus sp*. and *Bifidobacterium sp*.^27^ increased after FMT in the *Effect* group. This fits well with the notion of lower SCFA in IBS patients, as reviewed by Camilleri.^28^

The abundance of *Akkermansia muciniphila* was significantly reduced at 12 months after FMT, a signal also reported in a recent case series.^29^ This species is known to have various effects on host T-cell immunology in mice, ^30^ and has been linked to a decrease in the integrity of the intestinal mucus layer.^9^ Furthermore, it has been shown to be among the five taxa associated with the production of 10% of fecal metabolites.^31^

### Growth rate analysis

In the *Effect* group at baseline, the heat map plot (Figure 4(a)) shows relatively poor growth rate for a series of Bacteroidetes species which are more active in the *Donor* feces, combined with a pattern of higher growth rates for a series of other species that are not active in the *Donor* group. At 12 months, however, the resulting growth rate pattern is a conglomerate of these two patterns. The recipients seem to have a combination of their own initial microbiome and the *Donor* microbiome, as also shown by both the taxonomic and functional OPLS analyses (Figures 3 and 6). To our knowledge, the only published data on imputed growth rates that are somewhat comparable to this study found a decreased growth rate of *Roseburia hominis* when comparing IBS to healthy controls.^11^ This signal could not be found in our data, possibly due to a different study design (repeated measurements vs. case-control). For the *No effect* group, a similar pattern of combined growth rate profiles could be seen, though involving much fewer bacterial species, and possibly leaving the overall impact on the recipient microbiome too weak to induce a clinical effect.

### Functional analysis

The involvement of certain functional subsystems raises interesting questions about the basic pathophysiology of IBS. When we consider the role of a FODMAP-reduced diet in IBS treatment, SCFA and carbohydrate signals are of special interest. FODMAPs are a group of short-chain carbohydrates hypothesized to induce symptoms, possibly by altered colonic fermentation of these compounds, leading to a change in the functional output, which then induces gut symptoms.^6,8^ A recent study found reduced levels of inflammatory cytokines, an altered gut microbiota profile, and reduced levels of short-chain fatty acids to be associated with symptom relief from a low-FODMAP diet.^32^ Here, we can demonstrate that relative to *Donor* stool, the fecal samples from IBS patients have a lower representation of carbohydrate-related subsystems. In the *Effect* group, increases in *D-Galacturonate and D-Glucuronate utilization, D-ribose utilization, L-rhamnose utilization, and Lactose and Galactose uptake and utilization* were observed after 12 months. The L-rhamnose pathway was recently reported to be among the top five microbial pathways associated with 53% of fecal metabolites found in 479 unrelated individuals.^31^ Thus, the functional output of our FMT treatment closes the gap to the *Donor* profile in the *Effect* group. This change is not seen in the *No effect* group. All other subsystem clusters show a tendency of convergence toward the *Donor* profile in both the *Effect* and *No effect* groups.

#### SCFA

Strains that are known SCFA producers were relatively poorly represented in the baseline samples, a finding also reported by Pozuelo *et al*. .^23^ On the functional level, the Acetyl-CoA fermentation pathway leading to production of the short-chain fatty acid butyrate^33^ was reduced at baseline.

#### Micronutrients and growth factors

The observed increase in menaquinone pathways after FMT has interesting implications for growth patterns, and for the bacterial environment, as menaquinones represent a major class of growth factors for several bacterial species in the human gut.^34^ Also, the functional subsystem involved in synthesis of folate was significantly more abundant, which is thought to enhance the growth of commensals such as *Lactobacillus* sp.^35^ Subsystems involved in inorganic sulfur assimilation, ^36^ transport of Zinc, Manganese,^37^ and uptake of heme also changed significantly after FMT. The acquisition of metal ions is an essential survival factor for all microorganisms, both in the general environment and in the human host.^38^

### Strengths and weaknesses

The strengths of this study are: Firstly, our study design allowed microbiome analysis in two dimensions: Comparison between donor and IBS, and over time after the FMT intervention enabling both the generation of an “IBS profile,” and a proof of concept that microbiome changes mirror symptom improvement. Secondly, deep metagenomic sequencing enables an analysis of both taxonomy and functional features of the microbiome, yielding a better understanding of these components in IBS pathophysiology. There are also some weaknesses to be addressed: The sample size is quite small, which reduces the statistical power of the analyses. Also, the exploratory nature of the study precludes any firm conclusions. Importantly the transient freezer failure (mentioned in Materials and Methods) may have induced changes in the microbiome profiles, as reported by Tedjo *et al*.^39^ However, the temperature rise was relatively short and affected all samples in a similar manner. Thus, the observed systematic changes in the recipient groups probably reflect a genuine shift. Further, the observed shift of microbial profile might also reflect changes in diet, which is known to affect the bacterial composition almost on a daily basis. However, diet registrations in the clinical study did not reveal any systematic changes in FODMAP intake during the course of the study, although these were only registered at two time-points. Our microbiome characterization only addresses the luminal microbiota, whereas mucosa-associated microbes may be quite important (16). We included non-constipated IBS patients with a broad age and BMI span in order to retain a high clinical translation value for the results. However, in the present data set, this may introduce added complexity to the results due to hormonal effects and weight-related differences in the gut microbiome. Finally, the clinical study used only two donors, resulting in a very tight clustering of the *Donor* samples. This is unlikely to reflect the actual variability found in “normal” microbiomes.

### Conclusions

In conclusion, we have found distinct differences in microbiome composition between IBS patients and controls (*Donor*s). Subjects who experienced symptom relief after FMT showed a more explicit convergence toward the *Donor* sample profiles when compared to subjects who had no effect of the intervention. This supports the hypothesis that intestinal dysbiosis is a key component in IBS pathophysiology, as well as the notion that FMT could be a possible treatment for IBS.

## Materials and methods

### Clinical study

The REFIT study was described in detail in the primary publication.^15^ In short, we performed a double-blind, randomized, placebo-controlled, parallel-group, single-center study. Included participants had IBS-D or IBS-M as defined by the ROME III criteria, were between 18 and 75 years old, and scored moderately to severely according to the IBS severity scoring system (IBS-SSS; score ≥175). We randomly assigned participants to active or placebo FMT (2:1). The transplant was either freshly processed and used the same day, or previously stored in a freezer. Each participant’s own feces served as placebo. A dose of 8 mg loperamide was administered orally 2 h before endoscopy, to help participants retain the transplant. The transplant (50–80 g of feces mixed with 200 mL of isotonic saline and 50 mL of 85% glycerol) was administered to the cecum by colonoscope. The primary endpoint was a symptom relief of more than 75 points, as assessed by IBS-SSS, 3 months after FMT. Stool samples were collected from *Donors* at baseline, and from participants at baseline and after 6 and 12 months.

### Sequencing

All samples were frozen without additives in 1.7 mL Eppendorf tubes at −40°C. At one point during storage, the freezer had a technical failure, during which the temperature of the samples may have briefly exceeded 0°C (duration ≤12 h).

Sequencing was performed at the Genomics Support Center Tromsø (GSCT) at UiT the Arctic University of Norway. DNA isolation was performed on a QIAcube instrument (QIAgen, Hilden, Germany) using a QIAamp Fast DNA Stool Mini Kit (QIAgen). DNA concentration was measured on a Qubit 3 instrument (Thermo-Fisher, Massachusetts, USA). Library preparation was done with Nextera XT DNA library kit (Illumina, USA) using an input of 1 ng DNA. The samples were then sequenced on an Illumina NextSeq550 instrument with a NextSeq 500/550 High output v2 kit (300 cycles) (Illumina), generating 2*150 base-pair reads. The sequencing generated a mean of 27 271 415 (range 2 933 179 − 74 426 851) reads per sample.

## In silico *analysis*

### Pre-processing

The quality of the sequence data was investigated using FastQC, ^40^ and the data were filtered using the default settings for AfterQC, ^41^ with additional trimming of the 15 first nucleotides of each read. Optical sequence duplicates were removed with the optical distance of 40 using Clumpify.^42^ Fastq Screen^40^ was used, together with the human genome (GRCh38), to filter host contamination. The unsynchronized PE sequence files were repaired using Repair.^42^

### Taxonomic profiling

Taxonomic assignments of the pre-processed sequence reads were performed using Kaiju version 1.6.2^43^ against the pre-indexed NCBI BLAST nr, including fungi and microbial eukaryotes as reference database, and with the Greedy mode (higher sensitivity but longer run-time) and default parameter settings. The taxonomic abundance data from each sample – as generated by the Kaiju classification of the sequence reads – were merged into an abundance matrix and imported into Phyloseq^44^ along with the corresponding metadata.

### Functional profiling

Functional assignments of the pre-processed sequence reads were performed using SUPER-FOCUS version 0.32,^45^ aligning reads against the pre-indexed DB_98 database with DIAMOND 0.9.14.^46^ The functional classification results obtained for each SEED (a categorization system which organizes gene functional categories into a hierarchy with five levels of resolution) subsystem level were imported into Phyloseq, and the prune_taxa function was applied to retain only subsystems with absolute abundance >0.01%. The data were transformed into fractional abundances for multivariate analysis and visualization in STAMP.^47^ Differential abundance analysis of the SEED subsystems between groups was calculated using DESeq2.^48^ Only functional subsystems with differential abundance at a cut off log2 fold change (FC) >0.5, and adjusted *p*-value (FDR) ≤0.05 were reported.

### Statistical analysis

The species diversity (alpha diversity) in all samples was explored for absolute abundance values, using observed taxa, Chao1, Shannon, and Simpson indexes, and plots were generated using the plot_richness function. Taxa not seen more than five times in at least 20% of the samples – in the total dataset – were removed using the filter_taxa function. Differential abundance analysis between samples from participants who responded to treatment after 12 months, and the corresponding baseline samples, was performed on the taxa remaining after abundance filtering the data with DESeq2.^48^ Differentially abundant species were identified at a log2FC cut off of <-2 to >2, and an FDR cutoff of <10^−4^.

The filtered abundance data were transformed to fractional abundances, and only taxa with a mean fractional abundance >10^−5^ were used. The inter-individual differences (beta diversity) were calculated using the Bray–Curtis dissimilarity index on relative abundance values and explored using nMDS ordination plots generated by the Phyloseq package in R. The taxa remaining after abundance filtering were agglomerated at each taxonomic rank, using the tax_glom function to generate taxonomic bar plots, and subjected to multivariate analysis. Finally, relative abundance plots were generated, using the filtered data, for phyla at a cut off >0.01 relative abundance per sample group.

#### Regression analysis

The data were log transformed and Pareto scaled. Multivariate analysis was carried out using SIMCA software (version 15.0.2. 5559; Sartorius AB, Umeå, Sweden). Supervised orthogonal partial least squares projection to latent structures (OPLS) was applied, to facilitate the interpretation of changes in the composition of bacterial species and functional group profiles along the time points (baseline, 6, and 12 months). The performance of the OPLS models was described by *R*^2^X_cum_, *R*^2^Y_cum,_ and Q^2^_cum_, where *R*^2^X_cum_ is the cumulative modeled variation in X. *R*^2^Y_cum_ is the amount of variation in X correlated to Y (response matrix), and Q^2^_cum_ is the cumulative predicted ability of the model. The validity and degree of overfitting of the OPLS models were assessed by conducting analysis of variance testing of cross-validated predictive residuals (CV-ANOVA). An obtained *P*-value lower than 0.05 was considered as an indication of a significant model. Variables were ranked according to their scaled regression coefficients from the OPLS models.

### Growth rate analysis

To calculate the growth rate of species identified in the samples, GRiD^18^ was run. The samples were mapped against the stool-specific database^49^ using a minimum genome coverage cutoff = 0.2, enabling reassignment of ambiguous reads using Pathoscope2.^50^ In total, this analysis reported 959 species for the 47 samples. Species that occurred <15 times in <30 samples were eliminated from the further analysis, that was carried out on the remaining 340 species. Statistical testing on the ratios produced by GRiD for each species, per sample, was performed using ANOVA, and the results were visualized in STAMP.^47^ Only species at FDR cut off <0.00005 were included (n = 29) in the heat map.

## Supplementary Material

Supplemental Material

## Data Availability

The metagenomic sequence data generated during the current study have been deposited in the European Nucleotide Archive (ENA) under the study accession number PRJEB36140.

## Clinical trial identifier

The study was registered in ClinicalTrials.gov (NCT02154867).

## References

[1] Enck P, Aziz Q, Barbara G, Farmer AD, Fukudo S, Mayer EA, Niesler B, Quigley EMM, Rajilić-Stojanović M, Schemann M, et al. Irritable bowel syndrome. Nat Rev Dis Prim. 2016;2(1):16014. doi:10.1038/nrdp.2016.14. 27159638 PMC5001845

[2] Marchesi JR, Adams DH, Fava F, Hermes GDA, Hirschfield GM, Hold G, Quraishi MN, Kinross J, Smidt H, Tuohy KM, et al. The gut microbiota and host health: a new clinical frontier. Gut. 2016;65(2):330–15. doi:10.1136/gutjnl-2015-309990. 26338727 PMC4752653

[3] Ford AC, Lacy BE, Talley NJ. Irritable bowel syndrome. N Engl J Med. 2017;376(26):2566–2578. doi:10.1056/NEJMra1607547. 28657875

[4] Crouzet L, Gaultier E, Del’Homme C, Cartier C, Delmas E, Dapoigny M, Fioramonti J, Bernalier-Donadille A. The hypersensitivity to colonic distension of IBS patients can be transferred to rats through their fecal microbiota. Neurogastroenterol Motil. 2013;25(4):e272–82. doi:10.1111/nmo.12103. 23433203

[5] Berg LK, Fagerli E, Martinussen M, Myhre A-O, Florholmen J, Goll R. Effect of fructose-reduced diet in patients with irritable bowel syndrome, and its correlation to a standard fructose breath test. Scand J Gastroenterol. 2013;48:936–943. doi:10.3109/00365521.2013.812139. 23834159

[6] Gibson PR, Shepherd SJ. Evidence-based dietary management of functional gastrointestinal symptoms: the FODMAP approach. J Gastroenterol Hepatol. 2010;25(2):252–258. doi:10.1111/j.1440-1746.2009.06149.x. 20136989

[7] Catassi G, Lionetti E, Gatti S, Catassi C. The low FODMAP diet: many question marks for a catchy acronym. Nutrients. 2017;9(3):292. doi:10.3390/nu9030292. PMC5372955 28300773

[8] Staudacher HM, Whelan K. The low FODMAP diet: recent advances in understanding its mechanisms and efficacy in IBS. Gut. 2017;66(8):1517–1527. doi:10.1136/gutjnl-2017-313750. 28592442

[9] Bennet SMP, Ohman L, Simren M. Gut microbiota as potential orchestrators of irritable bowel syndrome. Gut Liver. 2015;9:318–331. doi:10.5009/gnl14344. 25918261 PMC4413965

[10] Ahmad OF, Akbar A. Microbiome, antibiotics and irritable bowel syndrome. Br Med Bull. 2016;120(1):91–99. doi:10.1093/bmb/ldw038. 27737852

[11] Vich VA, Imhann F, Collij V, Jankipersadsing SA, Gurry T, Mujagic Z, Kurilshikov A, Bonder MJ, Jiang X, Tigchelaar EF, et al. Gut microbiota composition and functional changes in inflammatory bowel disease and irritable bowel syndrome. Sci Transl Med. 2018;10(472):eaap8914. doi:10.1126/scitranslmed.aap8914. 30567928

[12] Lee WJ, Lattimer LDN, Stephen S, Borum ML, Doman DB. Fecal microbiota transplantation: a review of emerging indications beyond relapsing clostridium difficile toxin colitis. Gastroenterol Hepatol (N Y). 2015;11:24–32. 27099570 PMC4836576

[13] Simrén M, Barbara G, Flint HJ, Spiegel BMR, Spiller RC, Vanner S, Verdu EF, Whorwell PJ, Zoetendal EG. Intestinal microbiota in functional bowel disorders: a Rome foundation report. Gut. 2013;62(1):159–176. doi:10.1136/gutjnl-2012-302167. 22730468 PMC3551212

[14] Kelly CR, Kahn S, Kashyap P, Laine L, Rubin D, Atreja A, Moore T, Wu G. Update on FMT 2015: indications, Methodologies, mechanisms and outlook. Gastroenterology. 2015;149(1):223–237. doi:10.1053/j.gastro.2015.05.008. 25982290 PMC4755303

[15] Johnsen PH, Hilpüsch F, Cavanagh JP, Leikanger IS, Kolstad C, Valle PC, Goll R, et al. Faecal microbiota transplantation versus placebo for moderate-to-severe irritable bowel syndrome: a double-blind, randomised, placebo-controlled. Lancet Gastroenterol Hepatol. 2017;1253:17–24. 10.1016/S2468-1253(17)30338-2 29100842

[16] Halkjær SI, Christensen AH, Lo BZS, Browne PD, Günther S, Hansen LH, Petersen AM. Faecal microbiota transplantation alters gut microbiota in patients with irritable bowel syndrome: results from a randomised, double-blind placebo-controlled study. Gut. 2018;67(12):2107–2115. doi:10.1136/gutjnl-2018-316434. 29980607

[17] Myneedu K, Deoker A, Schmulson MJ, Bashashati M. Fecal microbiota transplantation in irritable bowel syndrome: A systematic review and meta-analysis. United Eur Gastroenterol J. 2019;7(8):1033–1041. doi:10.1177/2050640619866990. PMC6794695 31662860

[18] Emiola A, Oh J. High throughput in situ metagenomic measurement of bacterial replication at ultra-low sequencing coverage. Nat Commun. 2018;9(1):4956. doi:10.1038/s41467-018-07240-8. 30470746 PMC6251912

[19] Labus JS, Hollister EB, Jacobs J, Kirbach K, Oezguen N, Gupta A, Acosta J, Luna RA, Aagaard K, Versalovic J, et al. Differences in gut microbial composition correlate with regional brain volumes in irritable bowel syndrome. Microbiome. 2017;5(1):49. doi:10.1186/s40168-017-0260-z. 28457228 PMC5410709

[20] Peter J, Fournier C, Keip B, Rittershaus N, Stephanou-Rieser N, Durdevic M, Dejaco C, Michalski M, Moser G, et al. Intestinal microbiome in irritable bowel syndrome before and after gut-directed hypnotherapy. Int J Mol Sci. 2018;19(11):3619. doi:10.3390/ijms19113619. PMC6274728 30453528

[21] Wang Z, Xu C-M, Liu Y-X, Wang X-Q, Zhang L, Li M, Zhu S-W, Xie Z-J, Wang P-H, Duan L-P, et al. Characteristic dysbiosis of gut microbiota of Chinese patients with diarrhea-predominant irritable bowel syndrome by an insight into the pan-microbiome. Chin Med J (Engl). 2019;132(8):889–904. doi:10.1097/CM9.0000000000000192. 30958430 PMC6595763

[22] Lo Presti A, Zorzi F, Del Chierico F, Altomare A, Cocca S, Avola A, De Biasio F, Russo A, Cella E, Reddel S, Calabrese E, et al. Fecal and mucosal microbiota profiling in irritable bowel syndrome and inflammatory bowel disease. Front Microbiol. 2019;10:1655. doi:10.3389/fmicb.2019.01655. 31379797 PMC6650632

[23] Pozuelo M, Panda S, Santiago A, Mendez S, Accarino A, Santos J, Guarner F, Azpiroz F, Manichanh C. Reduction of butyrate- and methane-producing microorganisms in patients with irritable bowel syndrome. Sci Rep. 2015;5(1):12693. doi:10.1038/srep12693. 26239401 PMC4523847

[24] Hugerth LW, Andreasson A, Talley NJ, Forsberg AM, Kjellström L, Schmidt PT, Agreus L, Engstrand L, et al. No distinct microbiome signature of irritable bowel syndrome found in a Swedish random population. Gut. 2020;69(6):1076-1084. doi:10.1136/gutjnl-2019-318717. PMC7282555 31601615

[25] Pittayanon R, Lau JT, Yuan Y, Leontiadis GI, Tse F, Surette M, Moayyedi P. Gut microbiota in patients with irritable bowel syndrome—a systematic review. Gastroenterology. 2019;157(1):97–108. doi:10.1053/j.gastro.2019.03.049. 30940523

[26] Duan R, Zhu S, Wang B, Duan L. Alterations of gut microbiota in patients with irritable bowel syndrome based on 16S rRNA-targeted sequencing: a systematic review. Clin Transl Gastroenterol. 2019;10(2):e00012. doi:10.14309/ctg.0000000000000012. 30829919 PMC6407812

[27] Parada Venegas D, De la Fuente MK, Landskron G, González MJ, Quera R, Dijkstra G, Harmsen HJM, Faber KN, Hermoso MA. Short chain fatty acids (SCFAs)-mediated gut epithelial and immune regulation and its relevance for inflammatory bowel diseases. Front Immunol. 2019;10:277. doi:10.3389/fimmu.2019.00277. 30915065 PMC6421268

[28] Camilleri M. Intestinal secretory mechanisms in irritable bowel syndrome-diarrhea. Clin Gastroenterol Hepatol. 2015;13(6):1051–1057. doi:10.1016/j.cgh.2014.07.020. 25041862 PMC4297594

[29] Cruz-Aguliar RM, Wantia N, Clavel T, Vehreschild MJGT, Buch T, Bajbouj M, Haller D, Busch D, Schmid R, Stein-Thoeringer C, et al. An open-labeled study on fecal microbiota transfer in irritable bowel syndrome patients reveals improvement in abdominal pain associated with the relative abundance of *akkermansia muciniphila*. Digestion. 2019;100:127–138. doi:10.1159/000494252. 30423561

[30] Ansaldo E, Slayden LC, Ching KL, Koch MA, Wolf NK, Plichta DR, Brown EM, Graham DB, Xavier RJ, Moon JJ, et al. Akkermansia muciniphila induces intestinal adaptive immune responses during homeostasis. Science. 2019;364(6446):1179–1184. doi:10.1126/science.aaw7479. 31221858 PMC6645389

[31] Visconti A, Le Roy CI, Rosa F, Rossi N, Martin TC, Mohney RP, Li W, de Rinaldis E, Bell JT, Venter JC, Nelson KE, et al. Interplay between the human gut microbiome and host metabolism. Nat Commun. 2019;10(1):4505. doi:10.1038/s41467-019-12476-z. PMC6776654 31582752

[32] Hustoft TN, Hausken T, Ystad SO, Valeur J, Brokstad K, Hatlebakk JG, Lied GA, et al. Effects of varying dietary content of fermentable short-chain carbohydrates on symptoms, fecal microenvironment, and cytokine profiles in patients with irritable bowel syndrome. Neurogastroenterol Motil. 2017;29(4):e12969. doi:10.1111/nmo.12969. 27747984

[33] Venegas DP, De La Fuente MK, Landskron G, González MJ, Quera R, Dijkstra G, Faber KN, Hermoso MA, et al. Short chain fatty acids (SCFAs)mediated gut epithelial and immune regulation and its relevance for inflammatory bowel diseases. Front Immunol. 2019;10:277. doi:10.3389/fimmu.2019.00277 PMC6421268 30915065

[34] Fenn K, Strandwitz P, Stewart EJ, Dimise E, Rubin S, Gurubacharya S, Clardy J, Lewis K. Quinones are growth factors for the human gut microbiota. Microbiome. 2017;5(1):161. doi:10.1186/s40168-017-0380-5. 29262868 PMC5738691

[35] Rossi M, Amaretti A, Raimondi S. Folate production by probiotic bacteria. Nutrients. 2011;3(1):118–134. doi:10.3390/nu3010118. 22254078 PMC3257725

[36] Carbonero F, Benefiel AC, Alizadeh-Ghamsari AH, Gaskins HR. Microbial pathways in colonic sulfur metabolism and links with health and disease. Front Physiol. 2012;3:448. doi:10.3389/fphys.2012.00448. 23226130 PMC3508456

[37] Weiss G, Carver PL. Role of divalent metals in infectious disease susceptibility and outcome. Clin Microbiol Infect. 2018;24(1):16–23. doi:10.1016/j.cmi.2017.01.018. 28143784

[38] Porcheron G, Garénaux A, Proulx J, Sabri M, Dozois CM. Iron, copper, zinc, and manganese transport and regulation in pathogenic Enterobacteria: correlations between strains, site of infection and the relative importance of the different metal transport systems for virulence. Front Cell Infect Microbiol. 2013;3(DEC):90. doi:10.3389/fcimb.2013.00090. PMC3852070 24367764

[39] Tedjo DI, Jonkers DMAE, Savelkoul PH, Masclee AA, Van BN, Pierik MJ, Penders J, et al. The effect of sampling and storage on the fecal microbiota composition in healthy and diseased subjects. PLoS One. 2015;10(5):e0126685. doi:10.1371/journal.pone.0126685. PMC4449036 26024217

[40] Wingett SW, Andrews S. FastQ screen: A tool for multi-genome mapping and quality control. F1000Research. 2018;7:1338. doi:10.12688/f1000research.15931.2. 30254741 PMC6124377

[41] Chen S, Huang T, Zhou Y, Han Y, Xu M, Gu J. AfterQC: automatic filtering, trimming, error removing and quality control for fastq data. BMC Bioinform. 2017;18(S3):80. doi:10.1186/s12859-017-1469-3. PMC5374548 28361673

[42] BBMap download | SourceForge.net. https://sourceforge.net/projects/bbmap/. Accessed 276 2019.

[43] Menzel P, Ng KL, Krogh A. Fast and sensitive taxonomic classification for metagenomics with Kaiju. Nat Commun. 2016;7(1):11257. doi:10.1038/ncomms11257. 27071849 PMC4833860

[44] McMurdie PJ, Holmes S. phyloseq: an R package for reproducible interactive analysis and graphics of microbiome census data. PLoS One. 2013;8:e61217. doi:10.1371/journal.pone.0061217. 23630581 PMC3632530

[45] Silva GGZ, Green KT, Dutilh BE, Edwards RA. SUPER-FOCUS: a tool for agile functional analysis of shotgun metagenomic data. Bioinformatics. 2016;32(3):354–361. doi:10.1093/bioinformatics/btv584. 26454280 PMC4734042

[46] Buchfink B, Xie C, Huson DH. Fast and sensitive protein alignment using DIAMOND. Nat Methods. 2015;12:59–60. doi:10.1038/nmeth.3176. 25402007

[47] Parks DH, Tyson GW, Hugenholtz P, Beiko RG. STAMP: statistical analysis of taxonomic and functional profiles. Bioinformatics. 2014;30(21):3123–3124. doi:10.1093/bioinformatics/btu494. 25061070 PMC4609014

[48] Love MI, Huber W, Anders S. Moderated estimation of fold change and dispersion for RNA-seq data with DESeq2. Genome Biol. 2014;15:550. doi:10.1186/s13059-014-0550-8. 25516281 PMC4302049

[49] GRiD stool microbes database. ftp://ftp.jax.org/ohlab/GRiD_environ_specific_database/. Accessed 268 2019.

[50] Hong C, Manimaran S, Shen Y, Perez-Rogers JF, Byrd AL, Castro-Nallar E, Crandall KA, Johnson WE. PathoScope 2.0: a complete computational framework for strain identification in environmental or clinical sequencing samples. Microbiome. 2014;2(1):33. doi:10.1186/2049-2618-2-33. 25225611 PMC4164323

