# Supplementary material for: Effects of fecal microbiota transplantation in subjects with irritable bowel syndrome are mirrored by changes in gut microbiome
Source: Gut Microbes. 2020 Sep 29;12(1):1794263. doi: 10.1080/19490976.2020.1794263 (PMC7583512; doi:10.1080/19490976.2020.1794263)
Supplement: Supplemental Material [file KGMI_A_1794263_SM9522.zip › Supplementary information/Supplementary figures legends.docx]

Figure S1. Differential abundance of bacterial species with absolute abundance > 0.01% at phylum and species level in the *Effect* group, 12 months vs. baseline. Positive log2FC indicate enriched taxa after FMT, negative log2FC indicate decreased taxa after FMT. Species indicated on the X-axis are colored according to phyla. FDR cut off for inclusion in the plot was < 0.05.

Figure S2. Changes in level 1 functional subclasses. Panel A: Each participant profile at baseline was compared to the profile of the *Donor* transplant given. Panel B: The changes in profiles from baseline to 12 months following FMT. The error bars represent standard errors of mean (SEM).

Figure S3. Changes in level 3 functional subclasses within the Carbohydrates cluster, from baseline to 12 months, following FMT. The error bars represent standard errors of mean (SEM).
