# Supplementary figures and images for: Effects of fecal microbiota transplantation in subjects with irritable bowel syndrome are mirrored by changes in gut microbiome
Source: Gut Microbes. 2020 Sep 29;12(1):1794263. doi: 10.1080/19490976.2020.1794263 (PMC7583512; doi:10.1080/19490976.2020.1794263)

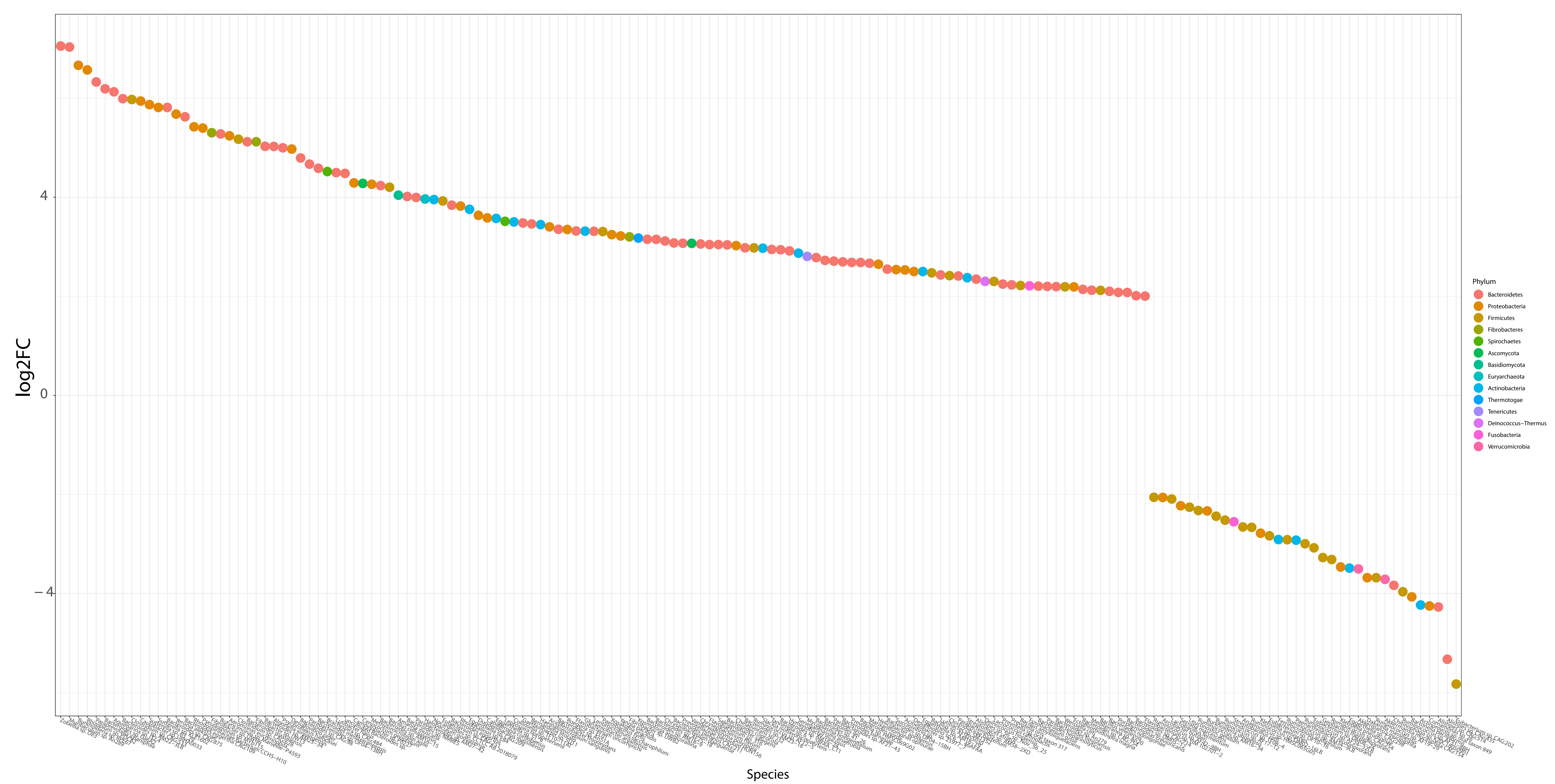

Supplement: Supplemental Material [file KGMI_A_1794263_SM9522.zip › Supplementary information/Figure_S1.pdf]

A

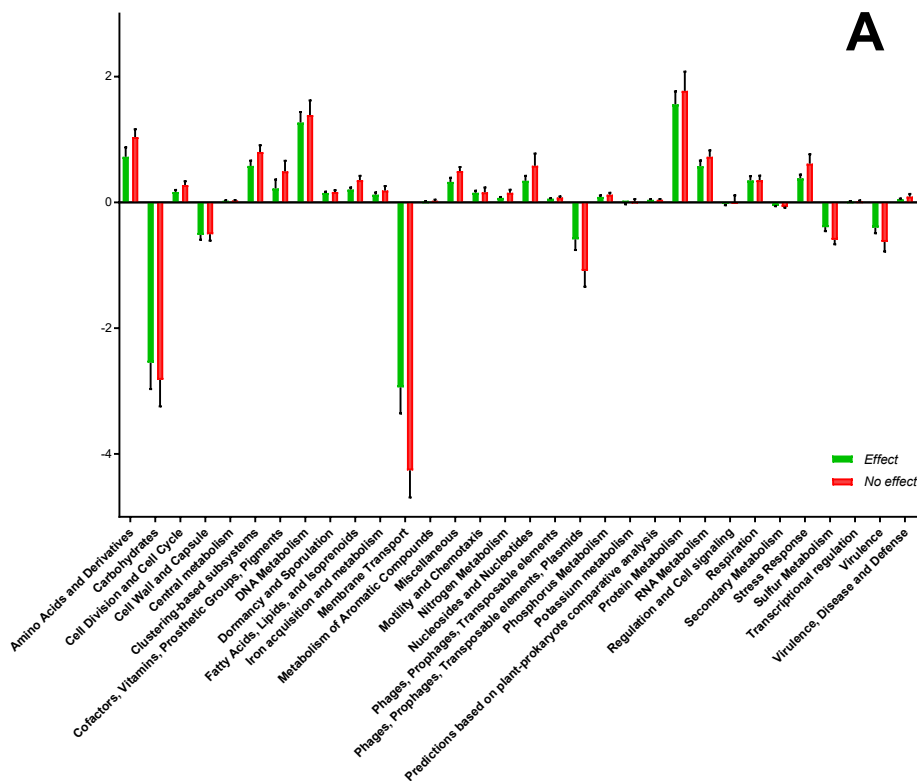

B

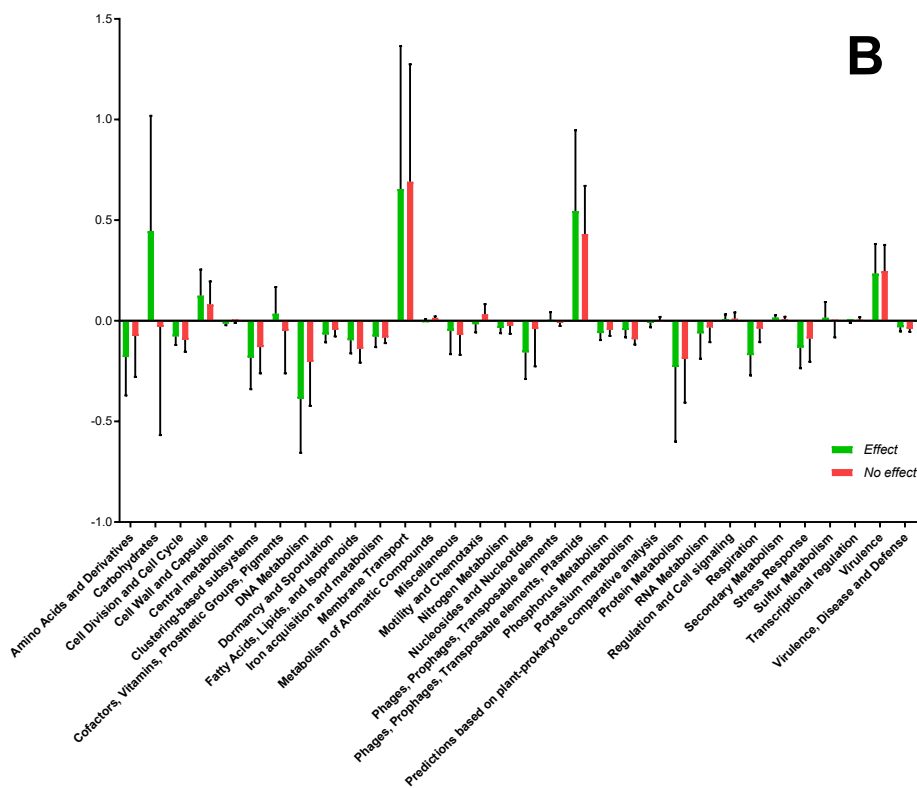

Supplement: Supplemental Material [file KGMI_A_1794263_SM9522.zip › Supplementary information/Figure_S2.pdf]

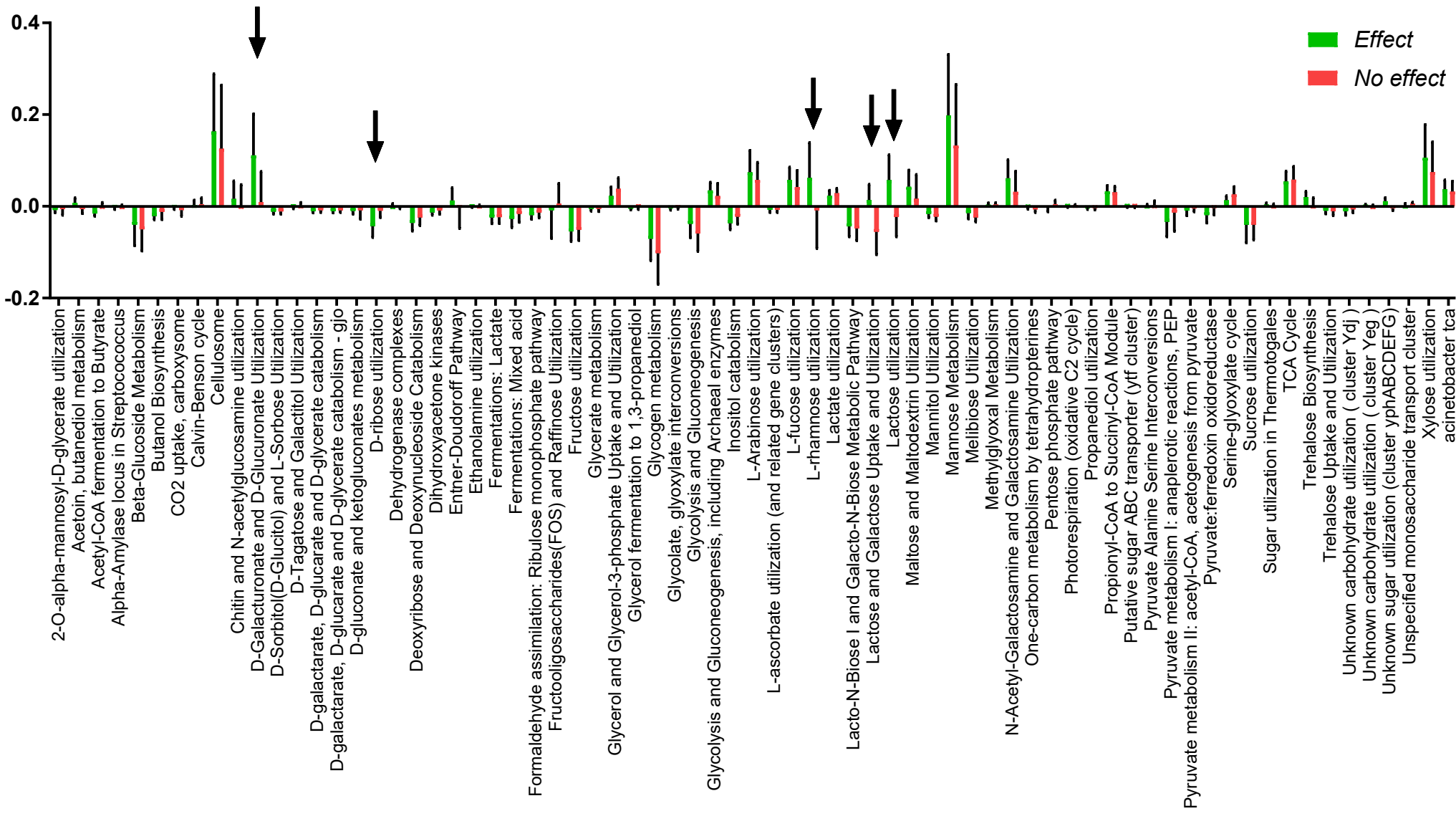

Supplement: Supplemental Material [file KGMI_A_1794263_SM9522.zip › Supplementary information/Figure_S3.pdf]
